# Downregulation of the E_2_ Subunit of 2-Oxoglutarate Dehydrogenase Modulates Plant Growth by Impacting Carbon–Nitrogen Metabolism in *Arabidopsis thaliana*

**DOI:** 10.1093/pcp/pcab036

**Published:** 2021-03-08

**Authors:** Jorge A Condori-Apfata, Willian Batista-Silva, David Barbosa Medeiros, Jonas Rafael Vargas, Luiz M Lopes Valente, Jorge Luis Pérez-Díaz, Alisdair R Fernie, Wagner L Araújo, Adriano Nunes-Nesi

**Affiliations:** Departamento de Biologia Vegetal, Universidade Federal de Viçosa, Viçosa, Minas Gerais 36570-900, Brazil; Departamento de Biologia Vegetal, Universidade Federal de Viçosa, Viçosa, Minas Gerais 36570-900, Brazil; Departamento de Biologia Vegetal, Universidade Federal de Viçosa, Viçosa, Minas Gerais 36570-900, Brazil; Max-Planck-Institute of Molecular Plant Physiology, Am Mühlenberg 1, Potsdam Golm 14476, Germany; Departamento de Biologia Vegetal, Universidade Federal de Viçosa, Viçosa, Minas Gerais 36570-900, Brazil; Departamento de Biologia Vegetal, Universidade Federal de Viçosa, Viçosa, Minas Gerais 36570-900, Brazil; Departamento de Biologia Vegetal, Universidade Federal de Viçosa, Viçosa, Minas Gerais 36570-900, Brazil; Max-Planck-Institute of Molecular Plant Physiology, Am Mühlenberg 1, Potsdam Golm 14476, Germany; Departamento de Biologia Vegetal, Universidade Federal de Viçosa, Viçosa, Minas Gerais 36570-900, Brazil; Departamento de Biologia Vegetal, Universidade Federal de Viçosa, Viçosa, Minas Gerais 36570-900, Brazil

**Keywords:** 2-OGDH, Primary metabolism, Respiration, TCA cycle

## Abstract

In *Arabidopsis thaliana*, two genes encode the E_2_ subunit of the 2-oxoglutarate dehydrogenase (2-OGDH), a multimeric complex composed of three subunits. To functionally characterize the isoforms of E_2_ subunit, we isolated Arabidopsis mutant lines for each gene encoding the E_2_ subunit and performed a detailed molecular and physiological characterization of the plants under controlled growth conditions. The functional lack of expression of E_2_ subunit isoforms of 2-OGDH increased plant growth, reduced dark respiration and altered carbohydrate metabolism without changes in the photosynthetic rate. Interestingly, plants from *e2-ogdh* lines also exhibited reduced seed weight without alterations in total seed number. We additionally observed that downregulation of 2-OGDH activity led to minor changes in the levels of tricarboxylic acid cycle intermediates without clear correlation with the reduced expression of specific E2-OGDH isoforms. Furthermore, the *e2-ogdh* mutant lines exhibited a reduction by up to 25% in the leaf total amino acids without consistent changes in the amino acid profile. Taken together, our results indicate that the two isoforms of E_2_ subunit play a similar role in carbon–nitrogen metabolism, in plant growth and in seed weight.

## Introduction

Mitochondrial metabolism plays important roles in many fundamental cellular processes, such as photosynthesis, photorespiration, nitrogen metabolism, redox regulation and signaling (Klodmann et al. 2011, Rao et al. 2017, Fuchs et al. 2020). Aerobic respiration is comprised of, at least, five complementary pathways, including (i) glycolysis, present in the cytosol, and to some extent, in the plastid, (ii) pyruvate oxidation, which occurs in the mitochondrial matrix by a multi-enzymatic complex called pyruvate dehydrogenase (PDH), which converts pyruvate into acetyl-CoA, (iii) the tricarboxylic acid (TCA) cycle, also located in the mitochondrial matrix, (iv) the electron transport chain, and (v) the oxidative phosphorylation, which takes place in the inner mitochondrial membrane (Fernie et al. 2004, van Dongen et al. 2011, Braun 2020). The TCA cycle is an universal feature of aerobic organisms and is composed by a set of eight enzymes primarily linking the condensation of oxaloacetate (OAA) and acetyl-CoA, with a series of oxidative steps, concomitantly releasing CO_2_ with the generation of reductants, such as NADH and FADH_2_, that support ATP synthesis and end with OAA regeneration (Sweetlove et al. 2010, Millar et al. 2011, Nunes-Nesi et al. 2013). The TCA cycle is embedded in a wider metabolic network that allows its activity to substantially contribute to other aspects of metabolism and provides carbon skeletons for a large number of biosynthetic processes (Sweetlove et al. 2010, Cavalcanti et al. 2014). The function of the TCA cycle in illuminated leaves still remains not fully understood and its operation in the light remains contentious (Nunes-Nesi et al. 2007b, Zhang et al. 2017, Zhang and Fernie 2018). In addition, the multiple alternative pathways of TCA cycle are unclear, but they may represent metabolic strategies that compensate for TCA inhibition under light. In general, in illuminated leaf, the respiratory rate is lower, while other metabolic processes, such as photosynthesis, photorespiration and nitrogen assimilation, are increased (Sweetlove et al. 2010, Tcherkez et al. 2012, Tcherkez et al. 2017).

Tomato succinate dehydrogenase (SDH) antisense lines (Araújo et al. 2011b) and SDH-deficient Arabidopsis plants (*Arabidopsis thaliana*) (Fuentes et al. 2011) displayed higher photosynthetic capacity as well as increased plant biomass. In addition, increased stomatal aperture and stomatal density were observed (Araújo et al. 2011b, Fuentes et al. 2011). Low expression of aconitase (ACO) in tomato plants altered the TCA cycle flux, reducing their intermediates, increasing adenylates levels and enhancing the photosynthetic rate (Carrari et al. 2003). In addition, decreases in mitochondrial malate dehydrogenase (mMDH) activity in tomato plants also increased photosynthetic capacity, total dry matter production and led to higher capacity of using l-galactono-lactone as a respiratory substrate (Nunes-Nesi et al. 2005). However, the loss of mMDH in *mmdh1mmdh2* Arabidopsis mutant plants affected photorespiration, increased respiration and led to slower growth due to decreased net CO_2_ assimilation (Tomaz et al. 2010). Interestingly, decreasing fumarase (FUM) activity via antisense inhibition led to a decreased photosynthesis and total plant biomass in tomato plants (Nunes-Nesi et al. 2007a). In Arabidopsis, two genes are found encoding for the mitochondrial isoform (FUM1) and the cytosolic isoform (FUM2) of fumarase, but only FUM1 appears to be essential for plant growth since attempts to obtain homozygous *fum1* knockout plants have failed (Pracharoenwattana et al. 2010). Measurements of apoplastic organic acids levels in SDH and fumarase antisense plants revealed a negative correlation between the levels of both malate and fumarate and stomatal conductance (Araújo et al. 2011a). In addition, reduction in mitochondrial citrate synthase (CS) activity resulted in a compromised nitrate assimilation without any effects on photosynthetic capacity or growth (Sienkiewicz-Porzucek et al. 2008). Similarly, reduced expression of succinyl-coenzyme A ligase did not affect photosynthetic performance (Studart-Guimarães et al. 2007). In Arabidopsis, it was shown that the NAD-dependent isocitrate dehydrogenase (IDH) activity is not limiting for TCA cycle activity and nitrogen assimilation. Nevertheless, it is important for the growth of liquid culture-grown mutants, which exhibited increased levels of citrate, hexose phosphate and higher sugar content (Lemaitre et al. 2007). In the same mutant lines for IDH, the levels of ammonium and free amino acids were moderately increased in comparison with wild-type plant cultures (Lemaitre et al. 2007). In tomato plants, mild reductions in IDH activity resulted in altered nitrate assimilation and pigmentation, without impacting plant growth (Sienkiewicz-Porzucek et al. 2010).

Hints to the regulation of the TCA cycle have been provided by in silico metabolic control analysis, which revealed that much of the control through this pathway resides in FUM, MDH and the 2-oxoglutarate dehydrogenase (2-OGDH) (Araújo et al. 2012c). The 2-OGDH complex occupies a central juncture in cellular metabolism linking the TCA cycle to nitrogen assimilation (Bunik and Fernie 2009, Araújo et al. 2013). Together with PDH, the 2-OGDH complex accounts for 11.2% of the total matrix volume (10.2% and 1.0%, respectively), which correspond to 49.0% and 4.7% of matrix protein volume, respectively (Fuchs et al. 2020).

2-OG may either be further converted in succinyl-CoA by 2-OGDH in the TCA cycle or used to provide carbon skeleton for inorganic nitrogen assimilation via glutamate biosynthesis (Araújo et al. 2013). Moreover, 2-OG is used as substrate in a range of oxidative reactions catalyzed by 2-OG-dependent dioxygenases (Kawai et al. 2014) and is involved in several important biochemical processes, including glucosinolates, flavonoid, alkaloid and gibberellin biosynthesis (Araújo et al. 2014, Farrow and Facchini 2014). 2-OG is also known to be an enzyme regulator, of enzymes such as cytosolic pyruvate kinase and PEP carboxylase, mitochondrial CS and the alternative oxidase, all of which are involved in sugar oxidation and/or organic acid flux and redox control between cytosol and mitochondria (Hodges 2002). Furthermore, 2-OG has been proposed as a signal metabolite in plants (Feria Bourrellier et al. 2009). This proposed role is largely based on the analogy to the function of 2-OG in conjuncture with the plastidial PII protein in plants (Uhrig et al. 2009). This protein potentially regulates a small number of enzyme systems in plants, including plastidial acetyl-CoA carboxylase (Feria Bourrellier et al. 2010) and *N*-acetyl-glutamate kinase (Ferrario-Méry et al. 2006, Feria Bourrellier et al. 2009).

The 2-OGDH catalyzes the oxidative decarboxylation of 2-OG into succinyl-CoA, generating NADH by the sequential operation of three enzymes: 2-OGDH (E_1_ subunit), dihydrolipoamide succinyl transferase (E_2_ subunit) and dihydrolipoamide dehydrogenase (E_3_ subunit) (Millar et al. 1999), with the consecutive action of several cofactors, such as thiamin diphosphate, Mg^2+^, lipoic acid and FAD^+^ (Strumilo 2005, Bunik and Fernie 2009). 2-OGDH exists as a polymeric structure that comprises an E_2_ core, to which are attached E_1_ and E_3_ homodimers (Millar et al. 1999). The two E_3_ polypeptides found in potato mitochondria are shown to be associated with both 2-OGDH and PDH complexes (Millar et al. 1999). The predicted polypeptide that it encodes has been proposed to participate in the catalytic function of PDH, 2-OGDH, glycine decarboxylase complex and branched-chain 2-oxoacid dehydrogenase complex (Millar et al. 1999, Timm et al. 2015). Recently, it was demonstrated that E_2_/E_3_ subunits of 2-OGDH interact with IDH and succinyl-CoA ligase forming a bridge that connects enzyme complexes in the mitochondrial matrix (Zhang et al. 2017). The regulation of 2-OGDH include allosteric responses to second messengers and metabolic indicators, such as Ca^2+^, ATP/ADP, SH/-S-S (thiol/disulfide), NADH/NAD^+^ and acetyl-CoA/CoA (Strumilo 2005, Bunik and Fernie 2009). The regulation of the dimeric components E_1_ and E_3_ by their substrates and effectors includes co-operative interactions of the active site and allosteric regulations of E_1_ by the product of E_3_, NADH (Bunik and Fernie 2009). Allosteric effectors not directly involved in the reaction, such as AMP and Ca^2+^, exert their regulatory influence in a highly interactive manner, by increasing the E_1_ affinity to their substrate (Strumilo 2005). When the ratio of the 2-OGDH substrate and/or products promotes the excessive formation of the dihydrolipate intermediate, 2-OGDH catalyzes the oxidation of the later by molecular oxygen with the formation of superoxide anion radical and thiyl radical of the complex-bound lipoate (Bunik and Fernie 2009).

It has been demonstrated, via chemical inhibition of 2-OGDH in potato tubers, that this enzyme plays an important role in nitrogen assimilation as well as in amino acid metabolism (Araújo et al. 2008). In addition, the same inhibitor of 2-OGDH was used in *A. thaliana* leaves provoking reduced respiration rates, likely associated with unbalanced carbon–nitrogen metabolism and cell homeostasis (Araújo et al. 2012b). Recently, the physiological and molecular function of E_1_ subunit of 2-OGDH was revealed in *A. thaliana* (Condori-Apfata et al. 2019). We demonstrated that the two E_1_ isoforms are not functionally redundant in terms of plant growth. Moreover, *e1-ogdh* mutant lines exhibited a substantial reduction in both respiration and CO_2_ assimilation rates followed by increased levels of sucrose, malate and fumarate and reduced levels of nitrogen-containing compounds, such as chlorophylls and nitrate (Condori-Apfata et al. 2019). Moreover, the 2-OGDH antisense inhibition in tomato plants did not alter the photosynthesis or growth rate but resulted in altered respiration rate, indicating that 2-OGDH plays an important role in modulating fluxes from 2-OG to amino acid metabolism (Araújo et al. 2012c).

Despite these findings, up to now, no direct genetic studies on the role of *E2-OGDH* specific isoforms in plants have been presented. In the Arabidopsis genome, the E_2_ subunit of 2-OGDH is encoded by two genes. However, the physiological and metabolic functions of these isoforms remain unknown. Here, we investigated the function of the two genes encoding the E_2_ subunit of 2-OGDH in Arabidopsis to uncover their physiological and metabolic functions under controlled conditions in both autotrophic and heterotrophic tissues.

## Results

### Expression profile of genes encoding E_2_ subunit of 2-OGDH complex

In *A. thaliana*, the E_2_ subunit of 2-OGDH is encoded by two genes, *E2-OGDH1* (At4g26910) and *E2-OGDH2* (At5g55070). These genes encode two proteins of 463 and 464 amino acids, respectively, with 82.9% of identity between them. The proteins have two conserved domains, the lipoyl domain of the dihydrolipoyl acyltransferase component and the 2-oxoacid dehydrogenase acyltransferase domain. Using the subcellular localization database for Arabidopsis proteins (SUBA4, http://suba.live) (Heazlewood et al. 2007, Hooper et al. 2017), we verified that both *E2-OGDH1* and *E2-OGDH2* are predicted to be mitochondrial proteins.

We additionally performed a phylogenetic analysis of the E_2_ subunit of 2-OGDH and identified many clusters, one of them represented by the monocot plants and the other clusters by dicot species, including the order Brasicales containing *A. thaliana* and *Brassica napus* (Supplementary Fig. S1A). The amino acid sequence of *A. thaliana E2-OGDH1* (NP_849452) revealed 83% identity to *B. napus* (CDY36727), 71% to *Solanum lycopersicum* (XP_004244101), 67% to *Brachypodium distachyon* (XP_003562264) and 69% to *Zea mays* (XP_008668074). The amino acid sequence of *A. thaliana E2-OGDH2* (NP_200318) revealed 92% identity to *B. napus* (CDY36727), 71% to *S. lycopersicum* (XP_004244101), 68% to *B. distachyon* (XP_003562264) and 69% to *Z. mays* (XP_008668074).

To further characterize E_2_ subunit encoding genes, we determined the expression pattern of both genes in different organs and tissues of *A. thaliana* wild-type (Col-0) plants by qRT-PCR analysis (Supplementary Fig. S1B). To this end, the total RNA was isolated from leaves of different phenological stages, flowers, siliques, roots and seedlings. In addition, we analyzed the expression of E_2_ subunit encoding genes in guard cell-enriched epidermal fragments, leaf blades and midrib. *E2-OGDH2* exhibited higher expression in comparison to *E2-OGDH1* in flowers and siliques. In young leaf, mature leaf, senescent leaf, cauline leaf, root and seedling, the expression of *E2-OGDH1* and *E2-OGDH2* did not differed significantly. Similarly, the expression level of these genes was similar in leaf blade and midrib. However, in epidermal fragments, the expression of *E2-OGDH2* was significantly higher in comparison to *E2-OGDH1*.

### Reduction in E_2_ subunit of 2-OGDH expression increases plant growth

**Fig. 1:**
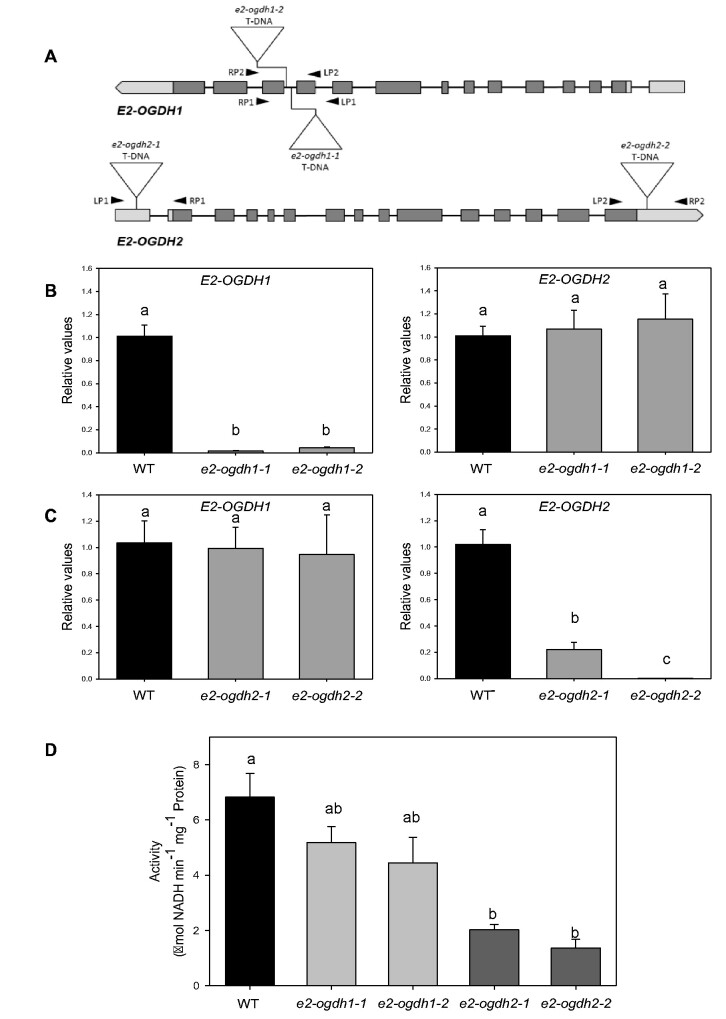
Expression and activity of 2-OGDH in Arabidopsis leaves of *E2-OGDH* mutant lines. (A) Schematic representation of the *E2-OGDH1* and *E2-OGDH2* gene structure. T-DNA insertion sites of the selected mutant lines are indicated. (B) Expression analysis *of E2-OGDH1* and *E2-OGDH2* in leaves of *e2-ogdh1-1* and *e2-ogdh1-2* mutant lines. (C) Expression analysis of *E2-OGDH1* and *E2-OGDH2* in leaves of *e2-ogdh2-1* and *e2-ogdh2-2* mutant lines. (D) 2-OGDH activity in fully expanded leaves of 5-week-old plants of *e2-ogdh1* and *e2-ogdh2* mutant lines. Values are presented as means ± SE of four individual plants. Different letters indicate a significant difference at *P *<* *0.05 using one-way ANOVA followed by post hoc Tukey's test, alpha = 0.001.

We next characterized at molecular and physiological levels the function of E_2_ subunit of 2-OGDH under controlled growth conditions. For this purpose, two mutant lines containing a T-DNA element inserted in *E2-OGDH1* gene were isolated and named *e2-ogdh1-1* and *e2-ogdh1-2*. Likewise, for the *E2-OGDH2* gene, we selected two T-DNA mutant lines named *e2-ogdh2-1* and *e2-ogdh2-2* (Fig. 1A).

The *e2-ogdh1-1* and *e2-ogdh1-2* mutant lines displayed a clear reduction in the expression of *E2-OGDH1* in comparison with wild-type levels, while the same mutant lines displayed similar expression levels of *E2-OGDH2* (Fig. 1B). The *e2-ogdh2-1* and *e2-ogdh2-2* mutant lines also displayed a clear reduction in the expression of the target gene *E2-OGDH2* in comparison with wild-type levels. Accordingly, the *e2-ogdh2-1* and *e2-ogdh2-2* mutant lines displayed similar expression levels of *E2-OGDH1* gene (Fig. 1C). We next assessed the 2-OGDH activity in mitochondrial enriched extracts from whole rosettes of the *e2-ogdh1* and *e2-ogdh2* mutant lines. This analysis revealed a clear reduction in the 2-OGDH activity of the e2-*ogdh2*-*1* and e2-*ogdh2-2* lines but only a minor and nonsignificant reduction in *e2-ogdh1-1* and *e2-ogdh1-2* plants in relation to wildtype (Fig. 1D).

**Fig. 2:**
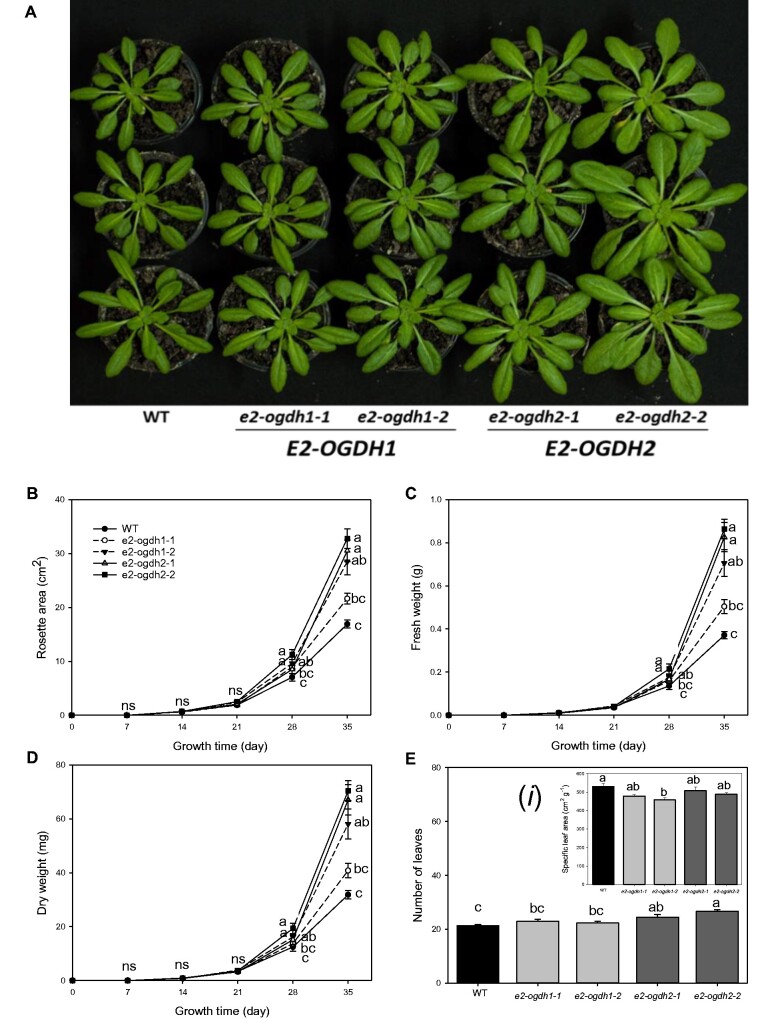
Growth phenotype of Arabidopsis *E2-OGDH* mutant plants. (A) *E2-OGDH1* and *E2-OGDH2* mutant plants after four weeks of cultivation. (B) Total leaf fresh weight, (C) total leaf dry weight, (D) rosette area, (E) number of leaves, and (Ei) specific leaf area. The lines used were as follows: the wild-type (WT, black bars); *E2-OGDH1* mutant lines (light gray bars); and *E2-OGDH2* mutant lines (dark gray bars). Values are presented as means ± SE of five individual plants per line. Different letters indicate a significant difference at *P *<* *0.05 using one-way ANOVA followed by post hoc Tukey's test, alpha = 0.05.

After confirming the *e2-ogdh* mutant lines for both genes, we grew all lines alongside one another under controlled growth conditions and determined morphological parameters of the genotypes. Four-week-old plants from *e2-ogdh2-1* and *e2-ogdh2-2* mutant lines appeared to have a faster development than wild-type plants (Fig. 2A). After 35 days, *e2-ogdh2-1*, *e2-ogdh2-2* and *e2-odgh1-2* mutant lines exhibited increases in the rosette area (Fig. 2B), and in the total leaf fresh and dry weight (Fig. 2C, D). Interestingly, an increase in the number of leaves was only observed in the *e2-ogdh2-1* and *e2-ogdh2-2* mutant lines in comparison to wild-type plants (Fig. 2E). The specific leaf area was significantly altered only in the *e2-ogdh1-2* mutant line compared to wild-type plants (Fig. 2Ei).

We next investigated the role of *E2-OGDH1* and *E2-OGDH2* in reproductive tissues by evaluating four yield related traits. Regarding the silique length, no significant difference was observed between the mutant lines and the wild-type plants (Supplementary Fig. S2A). Similarly, the number of seeds per silique and the total seed weight were unaltered in the mutant lines (Supplementary Fig. S2B, D). Surprisingly, the 1,000 seed weight was clearly reduced in *e2-ogdh1-2*, *e2-ogdh2-1* and *e2-ogdh2-2* plants (Supplementary Fig. S2C).

### Effects of the reduction in E_2_ subunit of 2-OGDH expression on seed germination and seedling establishment

**Fig. 3:**
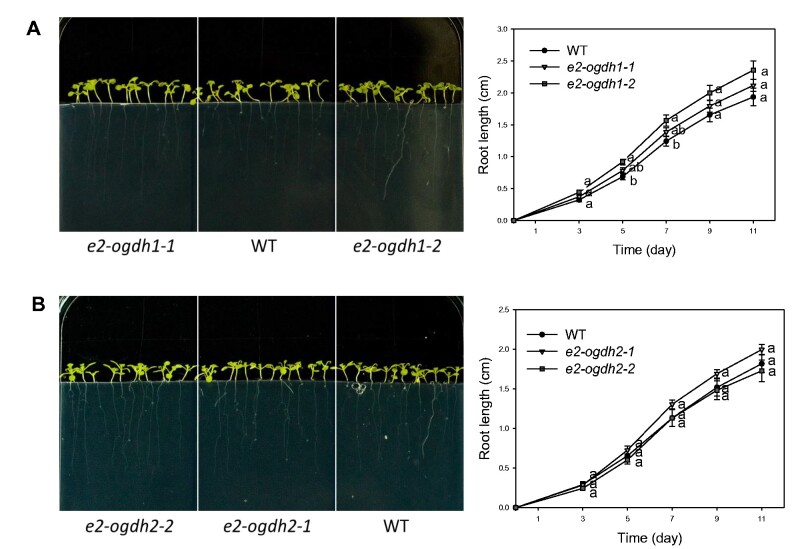
Root growth of seedlings from Arabidopsis *E2-OGDH* mutant lines. Seedling root growth of wild-type (WT), *E2-OGDH1* (A) and *E2-OGDH2* (B) mutant lines. The plants were grown on half-strength MS medium for 11 days after germination. Values are presented as means ± SE of four individual plates. Different letters indicate a significant difference at *P *<* *0.05 using one-way ANOVA followed by post hoc Tukey's test, alpha = 0.05.

To further investigate whether the reduction in *E2-OGDH* expression in *e2-ogdh* mutants affects both seed quality and viability, the rates of seed germination and seedling establishment were analyzed. On the first day in the light, seeds from *e2-ogdh1-2* mutant lines exhibited a significantly higher germination rate in comparison with wild-type seeds (Supplementary Fig. S3A). By contrast, by day 2, the germination rate in *e2-ogdh2-2* mutant line was significantly reduced when compared with wild-type seeds (Supplementary Fig. S3B). However, after 3 d in the light, we did not observe any significant change in seed germination of the *e2-ogdh* mutant lines in comparison to the wild-type plants (Supplementary Fig. S3). Interestingly, an increase in root length was observed in seedlings from the *e2-ogdh1-2* mutant line, specifically at 5 and 7 d after seed germination (Fig. 3A). By contrast, the *e2-ogdh2* mutant seedlings did not exhibit altered root growth in relation to the wild-type during 11 d after seed germination (Fig. 3B).

### Effects of the reduction in E_2_ subunit of 2-OGDH expression on photosynthetic parameters

**Fig. 4:**
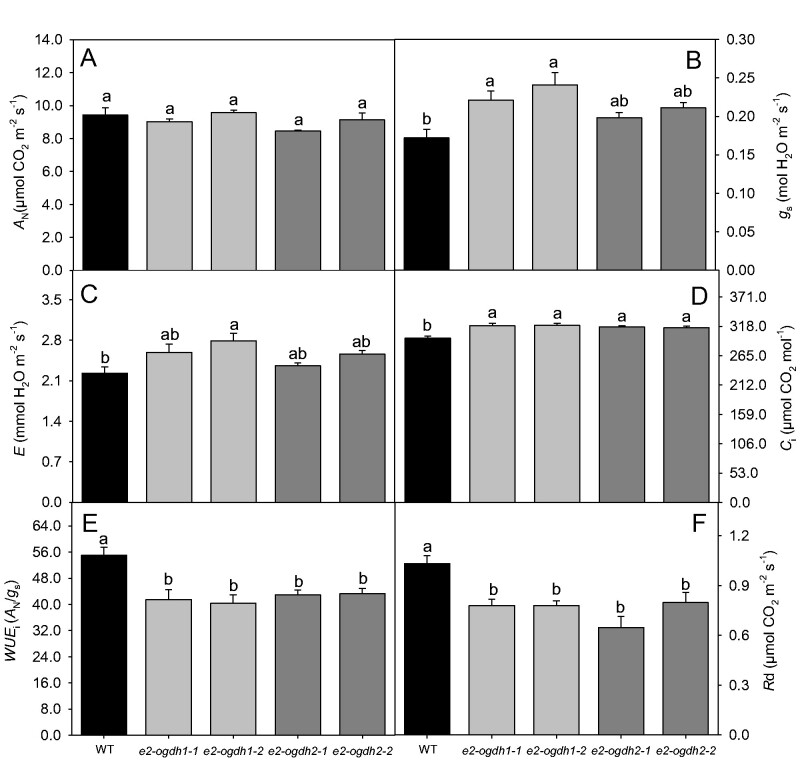
Effect of reduced expression of 2-OGDH E_2_ subunit on photosynthesis and respiration in fully expanded leaves of 4-week-old Arabidopsis plants under controlled growth conditions. (A) Net photosynthesis (*A*_N_); (B) stomatal conductance (*g*s); (C) transpiration rate (*E*); (D) internal CO_2_ concentration (*C_i_*); (E) intrinsic water-use efficiency (*WUEi*); and (F) dark respiration (*R*d). Instantaneous gas exchange analysis was performed after 1 h of illumination during the light period under 150 μmol photons m^−2^ s^−1^. Values are presented as means ± SE of five individual plants per line. Different letters indicate a significant difference at *P *<* *0.05 using one-way ANOVA followed by post hoc Tukey's test, alpha = 0.05.

We next performed a full characterization of photosynthetic capacity of *e2-ogdh* mutant lines. Overall, the net photosynthesis (*A*_N_) was not affected by the reduced expression of *E2-OGDH* genes under our controlled growth conditions (Fig. 4A). However, plants from *e2-ogdh1-1* and *e2-ogdh1-2* mutant lines exhibited an increased stomatal conductance (*g*_s_) as compared to wild-type plants (Fig. 4B). Regarding the transpiration rates (*E*), we observed an increase only in the *e2-ogdh1-2* mutant line (Fig. 4C). By contrast, the internal CO_2_ concentration (*C*i) was significantly higher in all mutant lines (Fig. 4D), which was associated with a clear decrease in the intrinsic water-use efficiency (*WUEi*) (Fig. 4E). We also performed measurements of fluorescence parameters under the same conditions. The *F*_v_/*F*_m_ ratio, which expresses the maximum PSII photochemical efficiency, was decreased only in *e2-ogdh2-1* mutant plants (Supplementary Table S1). Regarding the *F*_v_′/*F*_m_′ ratio, we did not observe significant changes between mutant lines and the wild-type plants. The other fluorescence-related parameters φPSII, NPQ, qP and ETR were also unaffected (Supplementary Table S1).

Subsequently, we carried out further gas exchange analyses under different light intensities ranging from 0 to 1,200 µmol photons m^−2^ s^−1^. These analyses revealed that plants from *e2-ogdh1-1* exhibit decreased *A*_N_ under 300, 600 and 800 µmol photons m^−2^ s^−1^. However, plants of *e2-ogdh1-2*, *e2-ogdh2-1* and *e2-ogdh2-2* displayed unaltered rates of *A*_N_ (Supplementary Fig. S4A). Similarly, no alteration was observed for *g*_s_ in plants under high light intensities (Supplementary Fig. S4B). We, furthermore, did not observe changes in either light saturation points (*I*_s_) or light compensation points (*I*_c_) in the mutant plants (Supplementary Table S2). Regarding *A*_N_ at the saturating light intensity, a significant reduction was observed in the *e2-ogdh2-1* mutant line (Supplementary Table S2). Interestingly, none of the changes in the gas exchange parameters were related to differences in the stomatal density and stomatal index in any of the mutant lines (Supplementary Fig. S5).

Since we have previously demonstrated that chemical or genetic inhibition of the 2-OGDH activity affects respiration rates (Araújo et al. 2008, Araújo et al. 2012b, Araújo et al. 2012c), we next decided to evaluate this parameter in dark-adapted leaves via infrared gas exchange analyses. As expected, plants from all mutant lines exhibited a strong reduction in leaf dark respiration (*R_d_*; Fig. 4F).

### Effects of the reduction in E_2_ subunit of 2-OGDH expression in metabolite levels

**Fig. 5:**
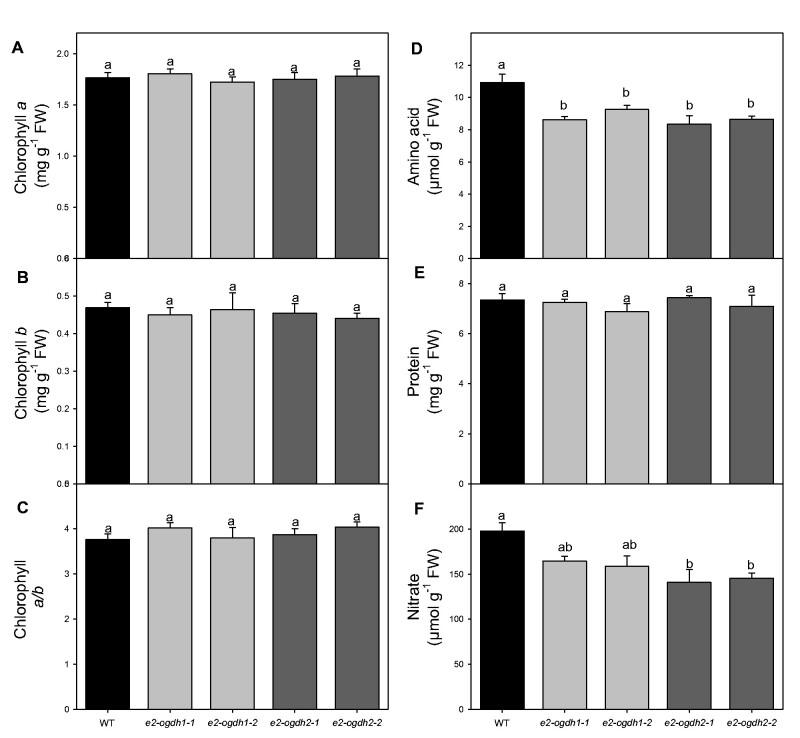
Effect of reduced expression of 2-OGDH E_2_ subunit on metabolite levels of the main nitrogen-containing compounds in fully expanded leaves of Arabidopsis. (A) Chlorophyll *a*; (B) chlorophyll *b*; (C) chlorophyll *a/b*; (D) total amino acids; (E) total soluble protein; and (F) nitrate. Metabolite levels were determined in 4-week-old fully expanded leaves harvested in the middle of the light period. Values are presented as means ± SE of five individual plants per line. Different letters indicate a significant difference at *P *<* *0.05 using one-way ANOVA followed by post hoc Tukey's test, alpha = 0.05.

Given the considerable changes in the *R_d_* and the recognized link between mitochondrial metabolism and carbon/nitrogen interactions (Nunes-Nesi *et al.* 2010), we further analyzed the levels of the main nitrogen-containing compounds. First, we quantified the photosynthetic pigments since these compounds have often been reported as important indicators of nitrogen deficiencies. These analyses revealed that, in leaves of *e2-ogdh1* and *e2-ogdh2* mutant lines, the levels of chlorophyll *a* and *b* were unaltered (Fig. 5A, B) as was the derived chlorophyll *a/b* ratio, when compared to wild-type plants (Fig. 5C). We next evaluated the total cellular amino acids and soluble protein levels as well as nitrate content in the same tissues. While the levels of total amino acids and nitrate were significantly reduced in all mutant lines (except for nitrate in leaves of *e2-ogdh1* lines; Fig. 5D, F), the level of total soluble protein remained unaltered in the mutant lines (Fig. 5E).

**Fig. 6:**
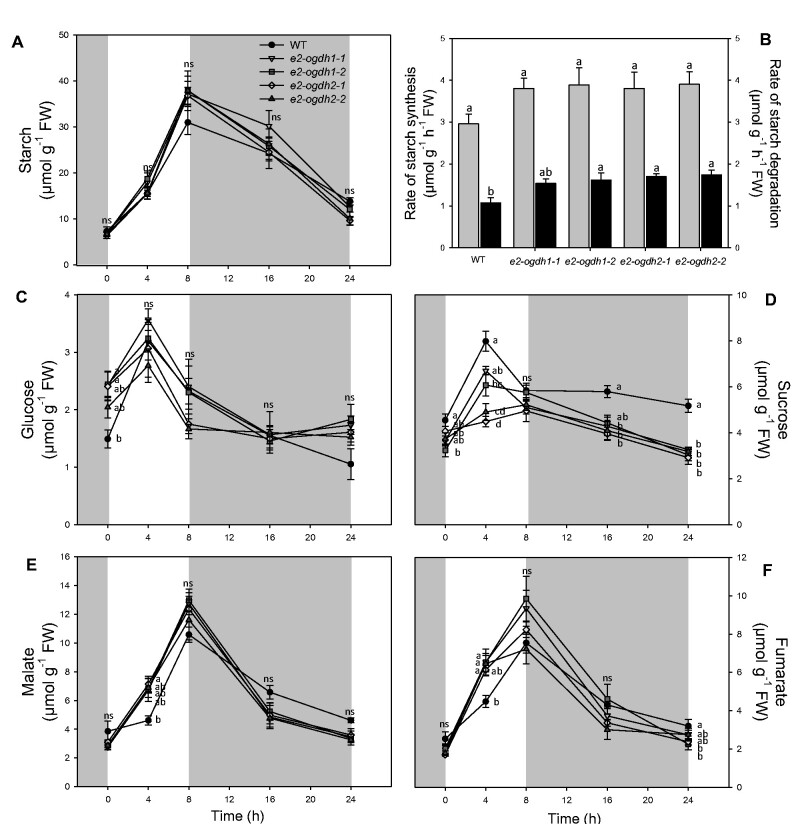
Diurnal changes of the main carbon related compounds in fully expanded leaves of Arabidopsis *E2-OGDH* mutant lines. (A) Starch content; (B) rates of starch synthesis (light gray bars) and starch degradation (black bars); (C) glucose; (D) sucrose; (E) malate; and (F) fumarate levels. Values are presented as means ± SE of five individual plants per line. Different letters indicate a significant difference at *P *<* *0.05 using one-way ANOVA followed by post hoc Tukey's test, alpha = 0.05.

We additionally quantified the main carbohydrates in leaves at different time points during the diurnal and dark cycle (Fig. 6). We determined the levels of starch, sucrose, glucose and organic acids malate and fumarate during the diurnal cycle (Fig. 6). In the light period, all mutant lines exhibited similar starch levels as compared to wild-type plants (Fig. 6A). At the end of the light and dark period, all mutant plants exhibited similar starch levels as compared to wild-type plants (Fig. 6A). We next calculated the rates of starch synthesis and degradation in leaves of all genotypes (Fig. 6B). Surprisingly, despite the nonsignificant changes in the starch accumulation at the end of the day and night, the rate of starch degradation was significantly increased in the mutant lines (with the exception of the line *e2-ogdh1-1*; Fig. 6B).

We also evaluated the levels of glucose, sucrose and organic acids (malate and fumarate) during the diel cicle. The levels of glucose were not consistently altered during the diurnal cycle in the mutant lines (Fig. 6C). By contrast, sucrose levels were significantly reduced in the middle of light period for the lines *e2-ogdh1-2*, *e2-ogdh2-1* and *e2-ogdh2-2* and also decreased at the end of the dark period in all mutant lines (Fig. 6D). The levels of malate were higher in leaves of the *e2-ogdh2-1* line at the middle of the light period and the levels of fumarate were increased in the lines *e2-ogdh1-1*, *e2-ogdh1-2* and *e2-ogdh2-2* also at the middle of the light period (Fig. 6E, F).

**Fig. 7:**
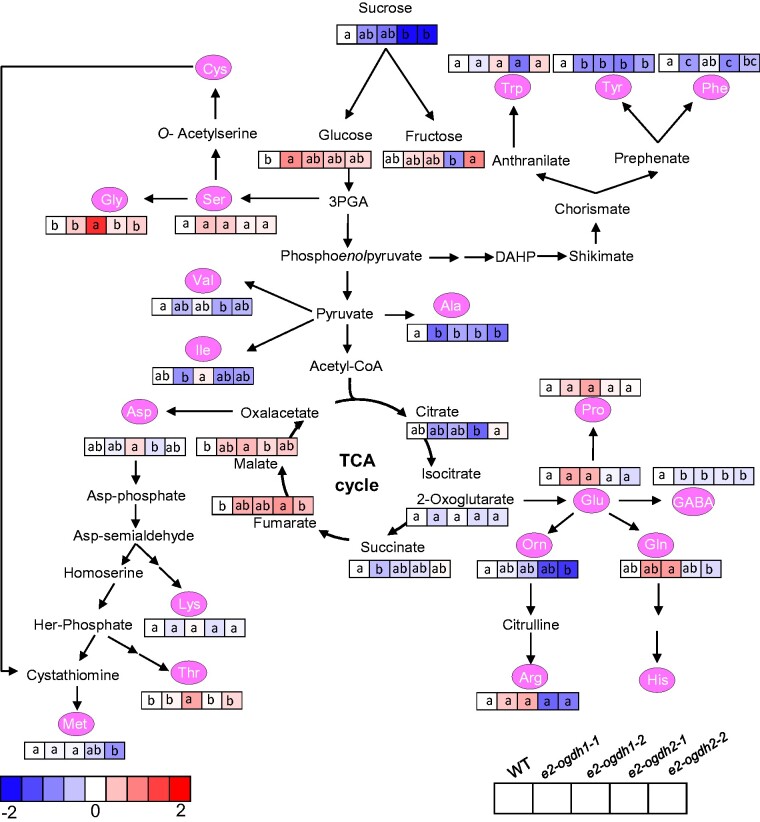
Relative metabolite content in fully expanded leaves of wild-type (WT) and *E2-OGDH* mutant lines at the middle of light period. The full data sets from these metabolic profiling studies are additionally available in Supplementary Table S3. Data are normalized with respect to the average response calculated for the corresponding WT (to allow statistical assessment, individual plants from this set were normalized in the same way). Different colors represent levels of metabolite fold change where red is increasing and blue is decreasing. Values are presented as means±SE of five individual plants per line. Different letters indicate a significant difference at *P *<* *0.05 using one-way ANOVA followed by post hoc Tukey's test, alpha = 0.05.

To have a broader overview of alterations in metabolite levels displayed by reduced expression of E2-OGDH isoforms, we next evaluated the relative metabolite levels in leaves using an established gas chromatography–mass spectrometry (GC–MS) approach for metabolite profiling (Lisec et al. 2006). The relative levels of the annotated metabolites are presented as a heatmap in Fig. 7 and the full data set in Supplementary Table S3. Noteworthy, similar to the results obtained from the diurnal cycle experiment in the middle of the light period (Fig. 6C, D), we verified reduced levels of sucrose in the mutant lines for the E2-OGDH2 isoform (*e2-ogdh2-1* and *e2-ogdh2-2*) and unaltered levels of glucose (with exception of an increase in the *e2-ogdh1-1 *line). Surprisingly, the reduced expression *of E2-OGDH1* and *E2-OGDH2* did not result in 2-OG accumulation in the mutant lines in comparison with wild-type plants, whereas the levels of the related metabolite gamma-aminobutyric acid (GABA) were up to 13.3% reduced in all mutant lines. The amino acids derived from 2-OG, proline, glutamine, glutamic acid and arginine remained unaltered in all mutant lines. On the other hand, a significant reduction in the levels of ornithine was observed in the *e2-ogdh2-2* mutant line compared with wild-type. In addition, we observed that the levels of alanine and tyrosine were strongly reduced in all mutant lines; methionine was reduced only in the *e2*-*ogdh2*-2 mutant line; and phenylalanine decreased in plants from the lines *e2-ogdh1-1*, *e2-ogdh2-1* and *e2-ogdh2-2*. We also verified that asparagine and isoleucine showed no significant alterations in all mutant lines, whereas valine exhibited reduction in the *e2-ogdh2-1 *line. The levels of lysine, serine and tryptophan were unaltered in all mutant lines. Threonine and glycine levels were increased only in the *e2-ogdh1-2* mutant line.

Interestingly, we did not observe consistent changes in the levels of organic acids. There was a reduction in the levels of succinate, significantly reduced in the *e2-ogdh1-1* mutant line. Similar to the results from our diurnal measurements (Fig. 6E, F), we also observed an increase in the levels of malate in the *e2-ogdh1-2* mutant line and fumarate in the *e2-ogdh2-1* mutant line.

## Discussion

The mitochondrial 2-oxoglutarate dehydrogenase (2-OGDH) is a multitasking enzyme present in the mitochondrial matrix. This enzyme is required for maintaining the metabolic fluxes through the TCA cycle further connecting this pathway with nitrogen metabolism (Nunes-Nesi et al. 2010, Sweetlove et al. 2010). In the present work, the reduced expression level of each *E2-OGDH* subunit gene did not affect the expression levels of the other gene encoding the same subunit (Fig. 1B, C), suggesting that the genes do not compensate one another at the expression levels. Interestingly, our results additionally suggest that the two isoforms of E2-OGDH might be able to partially compensate each other at the posttranscriptional levels since 2-OGDH activity was still observed in all mutant lines despite the lack in the expression of a specific E2-OGDH isoform (Fig. 1D). Nevertheless, at the activity level, it was verified that the total enzyme activity of 2-OGDH complex is affected (up to 75% of reduction) only in mutant lines lacking the E2-OGDH2 isoform (Fig. 1D). The decrease in E2-OGDH1 isoform expression was not able to significantly reduce the total activity of 2-OGDH. Stronger effects at the activity levels were observed in mutant lines lacking the E1-OGDH2 isoform of E1-subunit (Condori-Apfata et al. 2019). In this case, the total 2-OGDH activity was reduced by 82% in the mutant lines. It is important to note 2-OGDH complex account for 1.0% of the total volume of the mitochondrial matrix, corresponding to 4.7% of the matrix protein volume (Fuchs et al. 2020). In addition, it has been demonstrated that both subunits have different number of protein copies per mitochondrion with the E2-OGDH2 (At5g55070) being the most abundant one (58,419 copies per mitochondrion) followed by E1-OGDH2 (At5g65750; 56,609 copies per mitochondrion), E1-OGDH1 (At3g55410; 44,115 copies per mitochondrion) and E2-OGDH1 (At4g26910; 650 copies per mitochondrion) being considerably less abundant (Fuchs et al. 2020). Thus, we conclude that E2-OGDH1 plays a minor role in the total 2-OGDH activity in comparison with isoform E2-OGDH2 at least under the controlled growth conditions for Arabidopsis used in our experiments. Nevertheless, at the transcriptional level, differences in the expression exhibited by both E2 isoforms in most of the autothrophic tissues were not significant, with the exception of epidermal strips (Supplementary Fig. S1B). The discrepancy between the similar transcript levels observed in the present study (Supplementary Fig. S1B) and the reported protein abundances (Fuchs et al. 2020) might indicate that E2-OGDH1 protein is probably either rapidly turned over or the transcripts are not translated. Thus, the minor effect on 2-OGDH activity observed in the *e2-ogdh1* mutants is in agreement with the lower amounts of E2-OGDH1 protein recently reported (Fuchs et al. 2020). These results indicate that posttranscriptional mechanisms are most likely responsible for the regulation of the total 2-OGDH complex activity in autotrophic tissues.

A recent study demonstrated that both isoforms of E2-OGDH are able to interact with other TCA cycle enzymes forming large complexes in the mitochondrial matrix, named as Metabolon (Zhang et al. 2017). The E2-OGDH1 was shown to interact with IDH2 and IDH6 as well as the NADP^+^-dependent IDH (ICDH) proteins, whereas the E2-OGDH2 isoform interacts with IDH5 and IDH6 (Zhang et al. 2017). In addition, together with isoforms of IDH, SDH and PDC subunits, both E2-subunit isoforms from Arabidopsis were shown to interact with a signal transduction-related protein described as omega-amidase (At5g12040) (Zhang et al. 2018), also known to be associated with amino acid metabolism (Zhang and Marsolais 2014). Such interactions are able to form transient complexes allowing the regulation of metabolic pathway fluxes by dynamic association and/or dissociation between enzymes and complexes ([Bibr pcab036-B6897452][Bibr pcab036-B7690091]). Similar interactions might be modified in E2-OGDH-silenced plants and consequently the metabolic flux through TCA cycle may be altered. We have provided circumstantial evidence for the presence of an altered interaction between enzymes within the metabolon, which might explain the minor effects on metabolite levels and uncorrelated phenotypes in plants with reduced 2-OGDH expression and in vitro activity of this enzyme in the E2-OGDH mutant lines. In any case, we cannot rule out that the decreased activity of 2-OGDH can also lead to changes in the metabolites levels that could be also affected by the altered interactions. We also are not able to ascertain whether this lack of correlation between 2-OGDH activity and metabolic phenotypes can be explained by how the activity was measured since both subunits could have distinct kinetics parameters, specific allosteric regulations or other factors that could affect the activity differently to each subunit. Since we cannot formally exclude the possibility that this differential activity could also produce side effects elsewhere in metabolism, further comprehensive analyses are clearly required to understand the many facets of this complex metabolic phenotype. In addition, the interpretation of the results of such experiments needs to be made with caution.

As would be anticipated, based on previous reports (Araújo et al. 2012c, Araújo et al. 2008), lower expression of the E_2_ subunits leads to a decreased respiration rate, in a range of 25% for *E2-OGDH1* and 30% for *E2-OGDH2* (Fig. 4F). Similar results were also observed in leaves from Arabidopsis plants lacking the expression of *E1-OGDH1* and *E1-OGDH2* isoforms (approximately 35% and 30% reduction in respiration rates, respectively; Condori-Apfata et al. 2019). These results are in close agreement with metabolic control analysis for the TCA cycle enzymes indicating that much of the flux control through this pathway resides in 2-OGDH complex (Araújo et al. 2012a). Intriguingly, in this study and in our previous work on E1-OGDH isoforms (Condori-Apfata et al. 2019), we did not observed any clear correlation between the changes in total 2-OGDH activity and dark respiration in leaves. As afore mentioned, we hypothesize that altered expression of different isoforms of 2-OGDH complex culminated with changes in terms of protein copies per mitochondrion further altering complexes’ interactions within the metabolon, altering leaf respiration in the dark. However, on illuminated leaves, the TCA cycle reactions might be bypassed by cytosolic reactions and transporters allowing non-conventional cyclic fluxes trough the pathway (Sweetlove et al. 2010), which also explain the altered respiration rates without corresponding changes in 2-OGDH diurnal activity. While the precise mechanism responsible by this phenotype seems to be unclear from this study, it remains to be investigated whether the chemical or genetic inhibition of 2-OGDH complex is able to affect both the number of protein copies of each subunit in the mitochondrion and the flux through the TCA cycle, not only in 2-OGDH-related mutants but also in other mutants involved in mitochondrial respiration.

### E2-OGDH subunit is important for nitrogen metabolism in leaves

The TCA cycle also provides carbon skeletons to support biosynthetic processes, which include amino acids biosynthesis, and thus, it is involved in nitrogen assimilation (Noguchi and Yoshida 2008, Nunes-Nesi *et al.* 2010, Foyer *et al.* 2011, Szal and Podgórska 2012). Reduced activity of NAD-IDH in tomato and Arabidopsis plants displayed few changes in photosynthetic parameters yet altered carbon and nitrogen metabolism (Lemaitre *et al.* 2007, Sienkiewicz-Porzucek *et al.* 2010). Similar alterations were observed in tomato plants with reduced mitochondrial citrate synthase activity (Sienkiewicz-Porzucek et al. 2008). In agreement, the reduction in the expression of E2-OGDH subunit reduced the total amino acids levels (Fig. 5D) without changes in pigments (Fig. 5A–C) and total soluble protein content (Fig. 5E) in all mutant lines. However, only the *e2-ogdh2* mutant lines showed a significant decrease in the nitrate level (Fig. 5F). These results corroborate with previous studies supporting the importance of 2-OGDH activity for nitrogen metabolism in plants (Araújo et al. 2008, Araújo et al. 2013, Araújo et al. 2014). Nevertheless, the mechanisms by which 2-OGDH activity and 2-OG interact with nitrogen metabolism still remain not fully understood.

Previous studies demonstrated that genetic inhibition of 2-OGDH in tomato (Araújo et al. 2012c) and chemical inhibition in Arabidopsis leaves and potato tubers (Araújo et al. 2008, Araújo et al. 2012b) increased the levels of 2-OG and activated an alternative pathway supplying intermediates to TCA cycle. In the present study, the reduction in the expression of E_2_ subunit displayed unaltered levels of 2-OG and reduced levels of GABA (Fig. 7, Supplementary Table S3). Thus, based on reduced GABA levels, we hypothesize that the GABA shunt might be able to partially bypass the 2-OGDH complex and succinyl-CoA ligase and thus supplies succinate to maintain the electron transport chain activity, as previously suggested in tomato (Araújo et al. 2012c). In fact, the *e2-ogdh2* mutant lines showed a significant reduction in nitrate content, without any symptoms of nitrogen deficiency, followed by unaltered chlorophylls and total soluble protein levels (Fig. 5), and heterogeneous changes in individual amino acids (Fig. 7). Thus, the reduced levels of nitrate and total amino acids observed in leaves of the E2-OGDH2 mutant plants could be directly connected to the well-characterized involvement of 2-OGDH in providing carbon skeletons for nitrogen assimilation (Hodges 2002). In keeping with this hypothesis, the changes in the levels of specific amino acids (such as GABA) could be linked to the major changes in the activity of the 2-OGDH (Fig. 7, Supplementary Table S3), suggesting an alternative path to supply TCA cycle intermediates. However, since the changes in the levels of several amino acids were minor and ammonium as well as other nitrogen-containing compounds were not measured and could also be increased, further analyses, including double mutant lines, associated with metabolite flux analysis, will ultimately be required to support this hypothesis.

### E2 OGDH subunit and its role on photosynthesis and carbon metabolism

In agreement with the chemical inhibition of the 2-OGDH in heterotrophic tissue (Araújo et al. 2008) and in autotrophic tissue (Araújo et al. 2012b) and the antisense inhibition of 2-OGDH in tomato (Araújo et al. 2012c), the mutant plants of each gene encoding the E2-OGDH subunit displayed a considerably reduction in the respiration rate (Fig. 4F). Furthermore, the lack in the expression of the two genes encoding the same subunit did not consistently alter the rate of CO_2_ fixation (Fig. 4A, Supplementary Fig. S4A). Despite the fact that the levels of the main carbon-containing metabolites (starch and glucose) were unaltered in the middle of the light period, the sucrose levels were reduced in *e2-ogdh* mutant lines (Fig. 6D), suggesting either a high consumption or higher sucrose export to sink tissues. Interestingly, the reduction in sucrose level was coupled with the fact that these mutant lines also showed a minor increase in the rate of starch degradation at night (Fig. 6B). Together, the reduced dark respiration and unaltered CO_2_ assimilation rates suggest that reduced expression of E_2_ subunit of 2-OGDH in leaves promotes changes in the carbon balance that led to a faster and higher plant growth (Fig. 2). However, additional evidence should be provided to further support this hypothesis since the sucrose content could, in principle, also be affected by an energy deficiency at the end of the night and whether this is indeed the case needs to be tested.

### E1 and E2 OGDH subunits play similar physiological roles in Arabidopsis plants

Here, we focused on the question whether and how reduction in two isoforms of *E2-OGDH* subunit gene expression would affect the overall plant performance of *A. thaliana* under controlled growth conditions. In particular, our main goal was to establish whether changes in the expression of *E2-OGDH* isoforms might affect plant growth and development and how these alterations are affecting carbon and nitrogen metabolism. In addition, we investigated whether the two E_2_ isoforms are not functionally redundant in terms of plant growth. We observed that deficient expression of the genes encoding the E2-OGDH subunit resulted in changes in the central metabolism (Fig. 7), consequently leading into alterations in plant growth (Fig. 2) and seed weight (Supplementary Fig. S2C). In addition, we demonstrated that both isoforms of E_2_ subunit play relatively similar roles, contrasting from E1-OGDH subunit isoforms (Condori-Apfata et al. 2019) where the two E1-OGDH isoforms do not seem to play functionally redundant roles in Arabidopsis plant growth and development.

We further observed that silencing E2-OGDH isoforms lead to increases in the rosette area accompanied by a higher fresh and dry leaf weight (Fig. 2) accompanied by increased number of leaves, specifically for *e2-ogdh2* mutant lines. It is important to mention that the effects in these parameters can be related to the reduction in 2-OGDH activity, where *e2-ogdh2* mutant lines exhibited strong reductions in the activity of 2-OGDH, whereas the *e2-ogdh1* lines showed only a minor effect on the 2-OGDH activity (Fig. 1D). These results suggest that a reduction of up to 75% in the 2-OGDH activity results in significant effects on plant growth, indicating that *E2-OGDH2* expression might be associated with plant development as previously demonstrated in tomato (Araújo et al. 2012c). Intriguingly, distinct roles for each isoform of E_1_ subunit in the regulation of plant growth have been suggested (Condori-Apfata et al. 2019). Similar to E2-OGDH mutant lines, the reduction in 2-OGDH activity by silencing the E1-OGDH2 isoform of E_1_ subunit led to increased plant growth whereas the opposite was observed for the E1-OGDH1 isoform (Condori-Apfata et al. 2019). Further investigation is clearly required to fully understand this complex interaction between 2-OGDH subunits and the effects of them on the modulation of plant growth.

We additionally observed that lower expression of *E2-OGDH1* and *E2-OGDH2* isoforms neither resulted in an alteration in the number of seeds per silique or in silique length or in the total seed weight (Supplementary Fig. S2). However, *e2-ogdh1-2*, *e2-ogdh2-1* and *e2-ogdh2-2* mutants displayed the reduction of 1,000 seed weight (Supplementary Fig. S2). The decrease in this parameter is closely related to the decrease in the 2-OGDH activity (Fig. 1D). By contrast, the absence in the expression of *E1*-*OGDH1* genes in mutant lines with low 2-OGDH activity did not affect the individual seed weight or the total seed weight but decreased the silique length and the number of seeds per silique (Condori-Apfata et al. 2019). Therefore, it seems reasonable to suggest that 2-OGDH subunits play a role on growth and plant development. Nevertheless, a clear reduction in seed weight, especially in mutant plants for *E2-OGDH2*, was observed (Supplementary Fig. S2C). Collectively, these results suggest that the E_2_ subunit most likely does not play an essential role during gametophyte development, but it is rather required for seed maturation. Interestingly, seed germination, which requires reserves mobilization and an intensive catabolism, was not affected in the mutants with the lower expression of *E2-OGDH1* or *E2-OGDH2* (Supplementary Fig. S3).

## Conclusions

The lack of expression of the two genes encoding E2-OGDH subunit led to a reduction in dark respiration, without dramatic changes in the photosynthetic processes. In addition, reduced *E2-OGDH* gene expression have impacted plant growth through a metabolic rewiring, altering both carbon and nitrogen metabolism. Our results collectively indicate that E_2_ subunit plays an important regulatory role on the rate of respiration and, consequently, plant metabolism, growth and yield. However, the lack of expression of *E2-OGDH2* gene showed stronger alterations in the mutant plants, suggesting its greater importance in comparison to *E2-OGDH1* gene. Although the E_1_ and E_2_ subunits are parts of the 2-OGDH complex, each with specific functions, the low expression of the genes encoding each subunit has a significant impact on respiration. Therefore, we concluded that, both isoforms of E_2_ subunit have similar roles in plant growth, suggesting absence of compensatory effects at the transcriptional level, between the two isoforms unlike the isoforms of E_1_ subunit, where each isoform apparently has different functions.

Overall, our results revealed a complex and respiration-controlling role of E_2_ subunit of the 2-OGDH complex. Further functional characterization of these genes under sub-optimal growth conditions will likely help us identifying genes that control and regulate plant respiration as well as photosynthetates partitioning between shoots and roots. In addition, the identification of other genes involved in this response may provide new directions with potential biotechnological applications for modulating plant growth.

## Materials and Methods

### Plant material and growth conditions

The Columbia ecotype (Col 0) of *A. thaliana* was used as background for all mutant lines. T-DNA insertion lines for *E2-OGDH1* (At4g26910) and *E2-OGDH2* (At5g55070) genes encoding the E_2_ subunit of complex 2-OGDH were selected from the Salk collection (Salk Institute for Biological Studies, La Jolla, USA). For both genes, two lines were isolated: *e2-ogdh1-1* (Salk_005851) and *e2-ogdh1-2* (Salk_084620) for *E2-OGDH1* gene and *e2-ogdh2-1* (Salk_207269) and *e2-ogdh2-2* (Salk_054508) for *E2-OGDH2* gene. For genotyping the T-DNA insertion lines, leaves of each plant were collected separately and genomic DNA was extracted. PCR analyses were performed for the target gene using left primer (LP) and right primer (RP), and for T-DNA insertion, a T-DNA-specific left border primer (LBb1.3) was used. The primers used were: for *e2-ogdh1-1*, LP (CGACATACATCATTGGTCTAGGCACTA) and RP (TGATATGACATCTAACAACACTGCAGGTT); for *e2-ogdh1-2*, LP (GATCATACGTAAGTGCGACATACATCATT) and RP (TGATATGACATCTAACAACACTGCAGGTT); for *e2-ogdh2-1*, LP (CTGAGATGCTGTTTTAGATGGTTGCCT) and RP (CGAATCAAACACTAACCGAAGCAGAAG); for *e2-ogdh2-2*, LP (GATTCCTCTTCTCTGTGTATTGTATCCC) and RP (GTGTTGTATTGC TCCTTTGAAATCGTCCA); and primer specific for the T-DNA, LBb1.3 (GATTTTGCCGATTTCGGAACCACCAT). After initial screening, knockout lines were isolated and homozygous plants were selected for further analyses.

Seeds of homozygous plants were surface sterilized and incubated for 4 d at 4°C in the dark on agar plates containing half-strength MS medium (Murashige and Skoog 1962). Seeds were subsequently germinated and grown in vitro. After 10 d, the seedlings were transferred from plates to commercial substrate and grown in a growth chamber under the same conditions. Wild-type and mutant lines were grown under short-day conditions (8 h/16 h of light/dark) with an irradiance of 150 µmol photons m^−2^ s^−1^, 22 and 20°C in the light and dark, respectively and 60% of relative humidity.

### Growth analyses

Rosette area, total leaf fresh weight and total leaf dry weight were measured at 7-day intervals from 14 to 35 d after transplanting. The rosette area was measured by a digital image method using a scanner (Hewlett Packard Scanjet G2410) and, subsequently, the images were processed using ImageJ software (Schindelin et al. 2015). For root growth measurements, seeds were surface sterilized and placed on half-strength MS medium with 0.8% agar and incubated at 4°C for 48 h and then grown vertically in plates in a growth chamber at 22°C, 150 μmol photons m^−2^ s^−1^, in a 12/12-h light/dark photoperiod. The root length was measured with the AxioVision software (Carl-Zeiss, Jena) as previously described (Batista-Silva et al. 2019). For the phenotyping of reproductive tissues, the seedlings were transferred to commercial substrate and kept in a growth chamber at 22°C, 60% relative humidity and irradiance of 150 μmol photons m^−2^ s^−1^ with a photoperiod of 12/12-h light/dark.

Siliques were harvested and cleared with 0.2 N NaOH and 1% SDS (w/v) solution to remove pigments according to Yoo et al. (2012). The images of cleared siliques were analyzed for the length and number of seeds with a digital camera (Canon Powershot A650 IS) attached to a stereo microscope (Zeiss Stemi 2000-C), and the measurement of silique length was performed on the images using ImageJ software (Schindelin et al. 2015). Weight of 1,000 seeds and the total seed production were determined by weighing the seeds collected from at least six individual plants.

### Stomatal density and stomatal index

After 2 h of illumination in the light/dark cycle, leaf impressions were taken from the abaxial surface of the first fully expanded leaf with dental resin imprints (Berger and Altmann 2000). Nail polish copies were made using a colorless glaze (Von Groll et al. 2002). The images were taken with a digital camera (Axiocam MRc) attached to a microscope (Zeiss, model AX10). In addition, the measurements were performed on the images using AxionVision software (Carls-Zeiss, Jena). Stomatal density and stomatal index were determined in at least six fields of 0.09 mm^2^ per leaf from four different plants as previously described (Medeiros et al. 2017).

### Analysis of 2-OGDH enzyme activity

Crude mitochondrial extracts from leaves were prepared as previously described (Keech et al. 2005). The maximum leaf activity of 2-OGDH was determined exactly as described by Omena-Garcia *et al.* (2017).

### Gas exchange and chlorophyll a fluorescence measurement

Gas exchange parameters were determined simultaneously with chlorophyll *a* (Chl *a*) fluorescence measurements as previously described (Medeiros et al. 2017). The analysis was performed using an open‐flow infrared gas exchange analyzer system (LI‐6400XT; LI‐COR Inc., Lincoln, NE, USA) equipped with an integrated fluorescence chamber (LI‐6400‐40; LI‐COR Inc.). Instantaneous gas exchange analysis was measured after 1 h of illumination during the light period under 150 μmol photons m^−2^ s^−1^. The reference CO_2_ concentration was set at 400 μmol CO_2_ mol^−1^ air. Afterward, in a second set of plants, photosynthetic light–response curves (*A*/PPFD) were determined using ambient CO_2_ concentration (*C*_a_) of 400 μmol CO_2_ mol^−1^ and an initial photosynthetic photon flux density (PPFD) of 1,000 μmol photons m^−2^ s^−1^. PPFD was next increased to 1,100 and 1,200 μmol m^−2^ s^−1^ and thereafter decreased to 0 μmol m^−2^ s ^−1^ (step changes to 1,000, 800, 400, 200, 100, 50, 25, 10 and 0 μmol m^−2^ s^−1^) as previously described (Yin et al. 2009). Simultaneously, Chl *a* fluorescence parameters were obtained (Wong et al. 2012).

Following the obtained photosynthetic light–response curves, net CO_2_ assimilation rate saturated by light (*A*_PPFD_); light compensation point (*I_c_*) and light saturation point (*I_s_*) were estimated by using the Microsoft Excel^®^ spreadsheet available in http://landflux.org/Tools.php where the *A*/PPFD curves were fitted in a non-rectangular hyperbola model following Marshall and Biscoe (1980) and Thornley and Johnson (1990).

All measurements were performed using the 2-cm^2^ leaf chamber at 25°C, as well as a 0.5 stomatal ratio (amphistomatic leaves), and leaf‐to‐air vapor pressure deficit was kept at 1.2 kPa, and the amount of blue light was set to 10% PPFD to optimize the stomatal aperture. Dark respiration (*R*_d_) was measured using the same gas exchange system described above on the same leaves used for the determination of the photosynthetic parameters after at least 1 h of dark acclimation (Rodeghiero et al. 2007).

The initial fluorescence emission (*F*_0_) was measured by illuminating dark‐adapted leaves with a weak modulated measuring beam (0.03 μmol of photons m^−2^ s^−1^). A saturating white light pulse (8,000 μmol m^−2^ s^−1^) was applied for 0.8 s to obtain the maximum fluorescence (*F*_m_). Further Chl fluorescence-associated parameters were estimated as previously described (Genty *et al.* 1989, Logan *et al.* 2007).

### Metabolite profiling

The whole rosette was harvested at different time points along with the 8/16-h light/dark cycle at the beginning, middle and end of the light period. In addition, we harvested samples in the middle and end of the dark period. Rosettes were flash frozen in liquid nitrogen and stored at −80°C until further analyzes. The levels of sugars (sucrose, glucose, fructose and starch) in the leaves were determined exactly as previously described (Fernie et al. 2001) and malate and fumarate as previously detailed by Nunes-Nesi *et al.* (2007b). The rates of starch biosynthesis [(starch concentration at the end of the light period − starch concentration at the beginning of the light period)/the number of hours of light] and starch degradation [(starch concentration at the end of the light period − concentration starch at the end of the dark period)/number of dark hours] were calculated. Proteins were determined using an established procedure (Bradford 1976) and total amino acids as previously described (Yemm et al. 1955). Nitrate levels were quantified as described by Fritz *et al.* (2006) and photosynthetic pigments were determined exactly as indicated by Porra *et al.* (1989).

The metabolite profile was carried out in samples of whole rosette harvested at the middle of the light period and was performed by GC–MS exactly following the protocol previously described (Lisec et al. 2006). Peaks were manually annotated, and ion intensity was determined by the aid of TagFinder software (Luedemann et al. 2012), using a reference library from the Golm Metabolome Database (Kopka et al. 2005) and following the recommended reporting format (Fernie et al. 2011).

### Phylogenetic analysis

A total of 27 amino acids sequences, which were retrieved from the GenBank database applying the BLASTp algorithm using amino acids sequence of *E2-OGDH1* (At4g26910) and *E2-OGDH2* (At5g55070) genes as query, were used for phylogenetic reconstruction. The chosen sequences were aligned using *CLUSTAL W*, trimmed (matrix with a 510-amino-acid length) and used to infer the phylogeny based on the Maximum Likelihood method (MEGA version 5) (Tamura et al. 2011). The Jones-Taylor-Thornton model of substitution with gamma distribution (G) was selected as the best fitting model, applying the tool ‘Find best DNA/Protein Models’ available in the MEGA Package. The robustness of the phylogenetic tree was estimated by bootstrap analysis using 1,000 replications.

### Gene expression analysis

After 4 weeks of growth, leaf samples were collected and total RNA was isolated and purified using TRIzol^®^ reagent (Invitrogen Life Technologies) according to the manufacturer’s protocol. The RNA quality and integrity were monitored by spectrophotometer and by agarose gel electrophoresis. The total RNA was treated with DNase I to remove possible contaminating genomic DNA in the samples. Two micrograms of RNA were used as template for first-strand cDNA synthesis using ImProm-II™ Reverse Transcriptase (Promega) and an oligo (dT) primer. Quantitative real-time PCR (qRT-PCR) amplification of *E2*-*OGDH1* cDNA-specific sequence was performed with a forward primer (TGTCAAGGATGTTGTGGAGGATC) and a reverse primer (CGCAATACTCGGGAAACTGTAAAG). In the same way, qRT-PCR amplification of *E2-OGDH2* cDNA-specific sequence was performed with a forward primer (AGGTAAAACCATTACGGATACTGC) and a reverse primer (AAACTCAATACACCGATGCTTTCC). qRT-PCR amplification of the actin encoding gene of *A. thaliana* with a forward primer (CTTGCACCAAGCAGCATGAA) and a reverse primer (CCGATCCAGACACTGTACTTCCTT) served as a control to normalize the transcripts of all samples.

### Statistical analysis

The experiments were performed using a completely randomized design. The obtained data set was subjected to analysis of variance (ANOVA) followed by Tukey’s multiple comparisons test, using the R statistical package (R Core Team 2013).

## Supplementary Data

Supplementary data are available at PCP online.

## Funding

Conselho Nacional de Desenvolvimento Científico e Tecnológico (CNPq) [grant/award numbers 313534/2020-9 to A.N.-N. and 402511/2016-6 to W.L.A.]; Fundação de Amparo a Pesquisa do Estado de Minas Gerais (FAPEMIG) [grant/award number: CRA-RED-00053-16 to A.N.-N].

## Supplementary Material

pcab036_Supp
