# Supplementary material for: Downregulation of the E2 Subunit of 2-Oxoglutarate Dehydrogenase Modulates Plant Growth by Impacting Carbon–Nitrogen Metabolism in Arabidopsis thaliana
Source: Plant Cell Physiol. 2021 Mar 8;62(5):798–814. doi: 10.1093/pcp/pcab036 (PMC8484937; doi:10.1093/pcp/pcab036)
Supplement: pcab036_Supp [file pcab036_supp.zip › pcp-2020-e-00137-File009.docx]

**SUPPLEMENTAL TABLES**

**Table S1** Effect of reduced expression of 2-OGDH E_2_ subunit on chlorophyll fluorescence parameters in leaves of four weeks old Arabidopsis plants. Wild-type and mutant lines were grown under short-day conditions (8 h/16 h of light/dark) with an irradiance of 150 µmol photons m^-2^ s^-1^. Values are presented as means ± SE of five individual plants per line. Different letters indicate a significant difference at *P* < 0.05 using one-way ANOVA followed by posthoc Tukey's test, alpha = 0.05.

| **Parameters** | **WT** | | ***e2-ogdh1-1*** | | ***e2-ogdh1-2*** | | ***e2-ogdh2-1*** | | ***e2-ogdh2-2*** | |
| --- | --- | --- | --- | --- | --- | --- | --- | --- | --- | --- |
| *F*_v_/*F*_m_ | 0.781 ± 0.001 | a | 0.774 ± 0.003 | ab | 0.771 ± 0.003 | ab | 0.769 ± 0.003 | b | 0.778 ± 0.002 | ab |
| *F*_v_'/*F*_m_' | 0.513 ± 0.004 | a | 0.534 ± 0.005 | a | 0.519 ± 0.007 | a | 0.521 ± 0.006 | a | 0.522 ± 0.005 | a |
| ɸPSII | 0.179 ± 0.006 | a | 0.169 ± 0.003 | a | 0.180 ± 0.005 | a | 0.169 ± 0.004 | a | 0.168 ± 0.007 | a |
| NPQ | 1.83 ± 0.13 | a | 1.79 ± 0.09 | a | 1.88 ± 0.09 | a | 2.00 ± 0.09 | a | 1.75 ± 0.08 | a |
| qP | 0.350 ± 0.015 | a | 0.317 ± 0.007 | a | 0.348 ± 0.009 | a | 0.325 ± 0.011 | a | 0.323 ± 0.015 | a |
| ETR | 78.27 ± 2.80 | a | 74.09 ± 1.37 | a | 78.83 ± 2.07 | a | 73.82 ± 1.61 | a | 73.63 ± 3.25 | a |

Abbreviations: WT, wild-type; *F_v_*/*F_m_*, maximum PSII photochemical efficiency; *F_v_’/F_m_’*, actual PSII photochemical efficiency; ɸPSII, quantum yield of PSII; NPQ, non-photochemical quenching; qP, photochemical quenching; ETR, relative electron transport rate.

**Table S2** Effect of reduced expression of 2-OGDH E2 subunit on photosynthetic parameters derived from light-response curves.Values are presented as means ± SE of five individual plants per line. Different letters indicate a significant difference at *P* < 0.05 using one-way ANOVA followed by posthoc Tukey's test, alpha = 0.05.

| **Parameters** | **WT** | | | | ***e2-ogdh1-1*** | | | | ***e2-ogdh1-2*** | | | | ***e2-ogdh2-1*** | | | | ***e2-ogdh2-2*** | | | |
| --- | --- | --- | --- | --- | --- | --- | --- | --- | --- | --- | --- | --- | --- | --- | --- | --- | --- | --- | --- | --- |
| *A*_PPFD_ (µmol CO_2_ m ^-2^s^-1^) | 12.39 | ± | 0.22 | a | 10.60 | ± | 0.28 | a | 11.42 | ± | 0.53 | ab | 10.63 | ± | 0.31 | b | 11.29 | ± | 0.43 | ab |
| *I*c (µmol m^-2^ s^-1^) | 17.68 | ± | 1.33 | a | 16.10 | ± | 0.76 | a | 18.42 | ± | 2.73 | a | 14.49 | ± | 0.63 | a | 18.33 | ± | 2.36 | ab |
| *I*s (µmol m^-2^ s^-1^) | 618.90 | ± | 138.6 | a | 618.79 | ± | 112.27 | a | 541.53 | ± | 57.6 | a | 619.48 | ± | 53.5 | a | 531.17 | ± | 72.2 | ab |

Abbreviations: WT, wild-type; A_PPFD_, net CO_2_ assimilation rate saturated by light; *I*c, light compensation point; *I*s, light saturation point.

**Table S3** Relative metabolite content in fully expanded leaves of wild-type (WT) and E2-OGDH mutant lines at the middle of light period. Data were normalized with respect to the mean response calculated for the corresponding WT (to allow statistical assessment, individual plants from this set were normalized in the same way). Data are presented as means ± SE (n=5). Different letters indicate a significant difference at *P* < 0.05 using one-way ANOVA followed by posthoc Tukey's test, alpha = 0.05.

| **Amino acids** | **WT** | | | | ***e2-ogdh1-1*** | | | | ***e2-ogdh1-2*** | | | | ***e2-ogdh2-1*** | | | | ***e2-ogdh2-2*** | | | |
| --- | --- | --- | --- | --- | --- | --- | --- | --- | --- | --- | --- | --- | --- | --- | --- | --- | --- | --- | --- | --- |
| Alanine | 1.00 | ± | 0.11 | a | 0.43 | ± | 0.03 | b | 0.61 | ± | 0.03 | b | 0.57 | ± | 0.04 | b | 0.44 | ± | 0.05 | b |
| Aspartic acid | 1.00 | ± | 0.06 | ab | 0.91 | ± | 0.06 | ab | 1.23 | ± | 0.08 | a | 0.84 | ± | 0.14 | b | 0.97 | ± | 0.07 | ab |
| Arginine | 1.00 | ± | 0.21 | a | 1.36 | ± | 0.41 | a | 1.55 | ± | 0.38 | a | 0.42 | ± | 0.06 | a | 0.47 | ± | 0.05 | a |
| Asparagine | 1.00 | ± | 0.13 | a | 1.39 | ± | 0.35 | a | 1.47 | ± | 0.32 | a | 0.51 | ± | 0.08 | a | 0.58 | ± | 0.13 | a |
| Glycine | 1.00 | ± | 0.13 | b | 1.34 | ± | 0.22 | b | 3.12 | ± | 0.30 | a | 1.16 | ± | 0.17 | b | 1.31 | ± | 0.28 | b |
| Glutamine | 1.00 | ± | 0.17 | ab | 1.72 | ± | 0.33 | ab | 1.75 | ± | 0.30 | a | 0.87 | ± | 0.02 | ab | 0.81 | ± | 0.08 | b |
| Glutamic acid | 1.00 | ± | 0.15 | a | 1.58 | ± | 0.33 | a | 1.51 | ± | 0.25 | a | 0.94 | ± | 0.09 | a | 0.81 | ± | 0.06 | a |
| GABA | 1.00 | ± | 0.02 | a | 0.87 | ± | 0.02 | b | 0.86 | ± | 0.03 | b | 0.88 | ± | 0.02 | b | 0.85 | ± | 0.02 | b |
| Isoleucine | 1.00 | ± | 0.21 | ab | 0.54 | ± | 0.05 | b | 1.10 | ± | 0.16 | a | 0.55 | ± | 0.01 | ab | 0.58 | ± | 0.11 | ab |
| Lysine | 1.00 | ± | 0.06 | a | 0.87 | ± | 0.09 | a | 1.00 | ± | 0.05 | a | 0.92 | ± | 0.06 | a | 0.88 | ± | 0.06 | a |
| Methionine | 1.00 | ± | 0.10 | a | 0.95 | ± | 0.09 | a | 0.95 | ± | 0.09 | a | 0.78 | ± | 0.03 | ab | 0.54 | ± | 0.02 | b |
| Ornithine | 1.00 | ± | 0.28 | a | 0.91 | ± | 0.17 | ab | 0.73 | ± | 0.13 | ab | 0.36 | ± | 0.03 | ab | 0.32 | ± | 0.06 | b |
| Phenylalanine | 1.00 | ± | 0.06 | a | 0.57 | ± | 0.01 | c | 0.93 | ± | 0.11 | ab | 0.51 | ± | 0.04 | c | 0.67 | ± | 0.08 | bc |
| Proline | 1.00 | ± | 0.16 | a | 1.27 | ± | 0.18 | a | 1.62 | ± | 0.06 | a | 1.08 | ± | 0.23 | a | 1.02 | ± | 0.21 | a |
| Serine | 1.00 | ± | 0.06 | a | 1.38 | ± | 0.12 | a | 1.30 | ± | 0.07 | a | 1.07 | ± | 0.09 | a | 1.10 | ± | 0.09 | a |
| Threonine | 1.00 | ± | 0.09 | b | 1.07 | ± | 0.07 | b | 1.62 | ± | 0.06 | a | 1.03 | ± | 0.05 | b | 1.24 | ± | 0.07 | b |
| Tryptophan | 1.00 | ± | 0.21 | a | 0.87 | ± | 0.15 | a | 1.21 | ± | 0.20 | a | 0.46 | ± | 0.08 | a | 1.25 | ± | 0.25 | a |
| Tyrosine | 1.00 | ± | 0.10 | a | 0.53 | ± | 0.05 | b | 0.52 | ± | 0.05 | b | 0.48 | ± | 0.02 | b | 0.59 | ± | 0.15 | b |
| Valine | 1.00 | ± | 0.16 | a | 0.68 | ± | 0.02 | ab | 0.94 | ± | 0.11 | ab | 0.61 | ± | 0.03 | b | 0.67 | ± | 0.07 | ab |

| **Organic Acids** |  |  |  |  |  |  |  |  |  |  |  |  |  |  |  |  |  |  |  |  |
| --- | --- | --- | --- | --- | --- | --- | --- | --- | --- | --- | --- | --- | --- | --- | --- | --- | --- | --- | --- | --- |
| Citrate | 1.00 | ± | 0.06 | ab | 0.57 | ± | 0.13 | ab | 0.64 | ± | 0.09 | ab | 0.42 | ± | 0.10 | b | 1.07 | ± | 0.28 | a |
| 2-oxoglutarate | 1.00 | ± | 0.07 | a | 0.80 | ± | 0.03 | a | 0.97 | ± | 0.04 | a | 0.83 | ± | 0.04 | a | 0.86 | ± | 0.05 | a |
| Succinate | 1.00 | ± | 0.11 | a | 0.69 | ± | 0.06 | b | 0.80 | ± | 0.03 | ab | 0.85 | ± | 0.05 | ab | 0.94 | ± | 0.09 | ab |
| Ascorbate | 1.00 | ± | 0.18 | a | 0.77 | ± | 0.05 | a | 0.82 | ± | 0.10 | a | 0.75 | ± | 0.11 | a | 0.94 | ± | 0.16 | a |
| Malonate | 1.00 | ± | 0.08 | a | 0.81 | ± | 0.05 | a | 0.89 | ± | 0.04 | a | 0.81 | ± | 0.02 | a | 0.96 | ± | 0.02 | a |
| Malate | 1.00 | ± | 0.09 | b | 1.34 | ± | 0.04 | ab | 1.46 | ± | 0.05 | a | 1.23 | ± | 0.04 | b | 1.34 | ± | 0.02 | ab |
| Fumarate | 1.00 | ± | 0.07 | b | 1.55 | ± | 0.06 | ab | 1.54 | ± | 0.03 | ab | 1.77 | ± | 0.03 | a | 1.45 | ± | 0.04 | b |
| **Sugars** |  |  |  |  |  |  |  |  |  |  |  |  |  |  |  |  |  |  |  |  |
| Galactose | 1.00 | ± | 0.17 | a | 1.38 | ± | 0.18 | a | 1.26 | ± | 0.08 | a | 1.25 | ± | 0.17 | a | 1.27 | ± | 0.09 | a |
| Sucrose | 1.00 | ± | 0.09 | a | 0.54 | ± | 0.10 | ab | 0.45 | ± | 0.09 | ab | 0.21 | ± | 0.05 | b | 0.12 | ± | 0.02 | b |
| Glucose | 1.00 | ± | 0.08 | b | 1.89 | ± | 0.12 | a | 1.55 | ± | 0.08 | ab | 1.35 | ± | 0.13 | ab | 1.25 | ± | 0.12 | ab |
| Fructose | 1.00 | ± | 0.12 | ab | 1.22 | ± | 0.23 | ab | 1.30 | ± | 0.20 | ab | 0.53 | ± | 0.08 | b | 1.95 | ± | 0.58 | a |
| Isomaltose | 1.00 | ± | 0.28 | a | 0.84 | ± | 0.16 | a | 0.63 | ± | 0.13 | a | 0.92 | ± | 0.11 | a | 1.05 | ± | 0.18 | a |
| Rhamnose | 1.00 | ± | 0.04 | a | 0.85 | ± | 0.04 | a | 0.94 | ± | 0.02 | a | 0.99 | ± | 0.05 | a | 0.90 | ± | 0.04 | a |
| Raffinose | 1.00 | ± | 0.34 | a | 1.42 | ± | 0.23 | a | 1.20 | ± | 0.23 | a | 1.34 | ± | 0.19 | a | 1.75 | ± | 0.24 | a |
| Trehalose | 1.00 | ± | 0.07 | a | 1.18 | ± | 0.11 | a | 0.96 | ± | 0.13 | a | 1.20 | ± | 0.11 | a | 1.07 | ± | 0.09 | a |
| **Sugar alcohols** |  |  |  |  |  |  |  |  |  |  |  |  |  |  |  |  |  |  |  |  |
| Erythritol | 1.00 | ± | 0.04 | a | 0.83 | ± | 0.04 | b | 1.02 | ± | 0.04 | a | 0.93 | ± | 0.03 | ab | 0.94 | ± | 0.02 | ab |
| Inositol, myo | 1.00 | ± | 0.08 | a | 0.92 | ± | 0.07 | a | 0.88 | ± | 0.04 | a | 0.98 | ± | 0.08 | a | 0.95 | ± | 0.06 | a |
| **Other metabolites** |  |  |  |  |  |  |  |  |  |  |  |  |  |  |  |  |  |  |  |  |
| Glycerate | 1.00 | ± | 0.14 | a | 1.04 | ± | 0.06 | a | 1.46 | ± | 0.13 | a | 1.34 | ± | 0.12 | a | 1.37 | ± | 0.26 | a |
| Glycerol | 1.00 | ± | 0.09 | a | 0.76 | ± | 0.04 | a | 0.89 | ± | 0.05 | a | 0.96 | ± | 0.05 | a | 0.86 | ± | 0.04 | a |
| Spermidine | 1.00 | ± | 0.11 | ab | 1.13 | ± | 0.09 | a | 1.08 | ± | 0.07 | ab | 1.15 | ± | 0.12 | a | 0.74 | ± | 0.04 | b |
| Putrescine | 1.00 | ± | 0.19 | ab | 1.07 | ± | 0.12 | a | 1.24 | ± | 0.07 | a | 0.93 | ± | 0.07 | ab | 0.57 | ± | 0.09 | b |
| Guanidine | 1.00 | ± | 0.18 | ab | 0.55 | ± | 0.04 | bc | 1.13 | ± | 0.13 | a | 0.45 | ± | 0.14 | c | 0.46 | ± | 0.09 | c |
| Lactate | 1.00 | ± | 0.14 | a | 0.87 | ± | 0.06 | a | 0.86 | ± | 0.12 | a | 0.91 | ± | 0.04 | a | 0.99 | ± | 0.10 | a |
