# Supplementary figures and images for: Downregulation of the E2 Subunit of 2-Oxoglutarate Dehydrogenase Modulates Plant Growth by Impacting Carbon–Nitrogen Metabolism in Arabidopsis thaliana
Source: Plant Cell Physiol. 2021 Mar 8;62(5):798–814. doi: 10.1093/pcp/pcab036 (PMC8484937; doi:10.1093/pcp/pcab036)

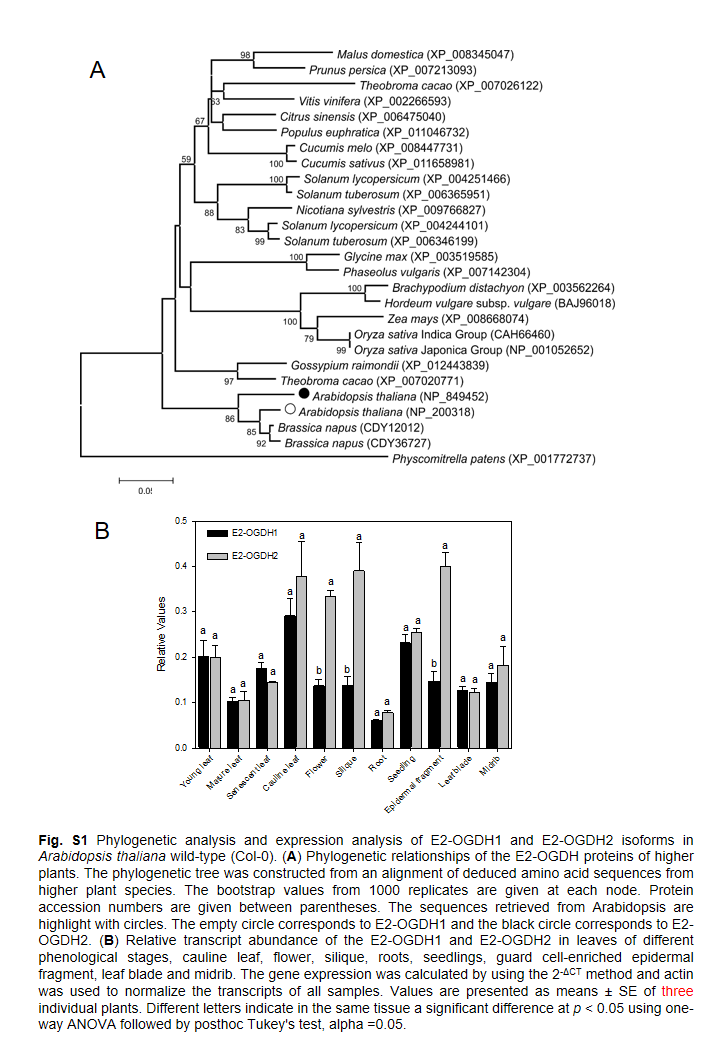

Supplement: pcab036_Supp [file pcab036_supp.zip › pcp-2020-e-00137-File010.tif]

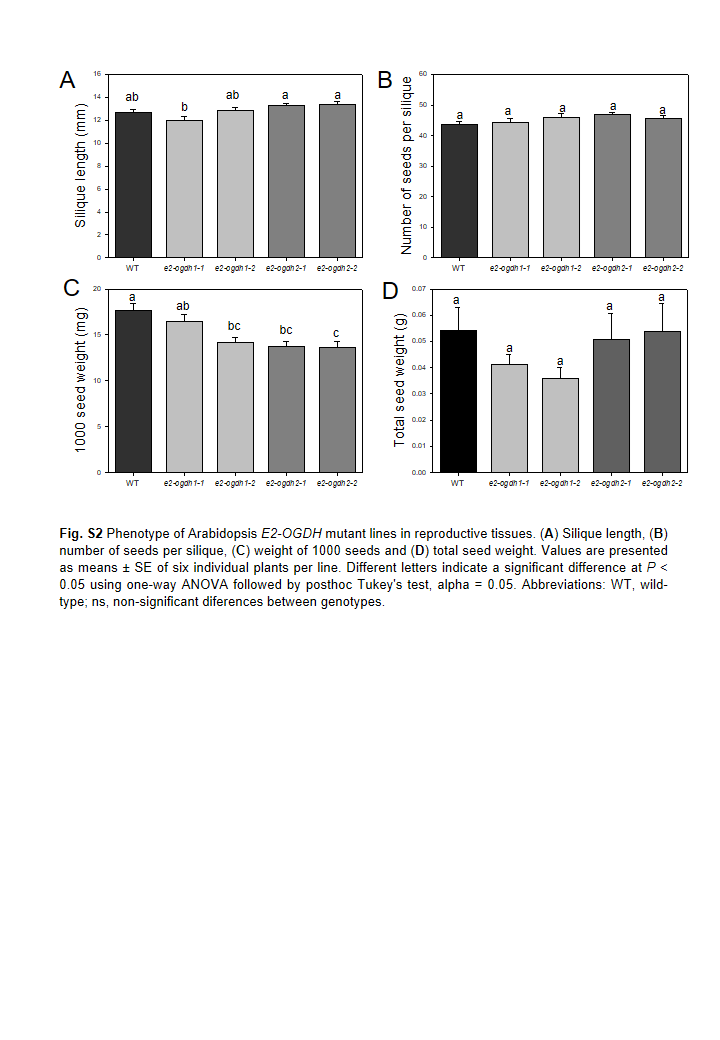

Supplement: pcab036_Supp [file pcab036_supp.zip › pcp-2020-e-00137-File011.TIF]

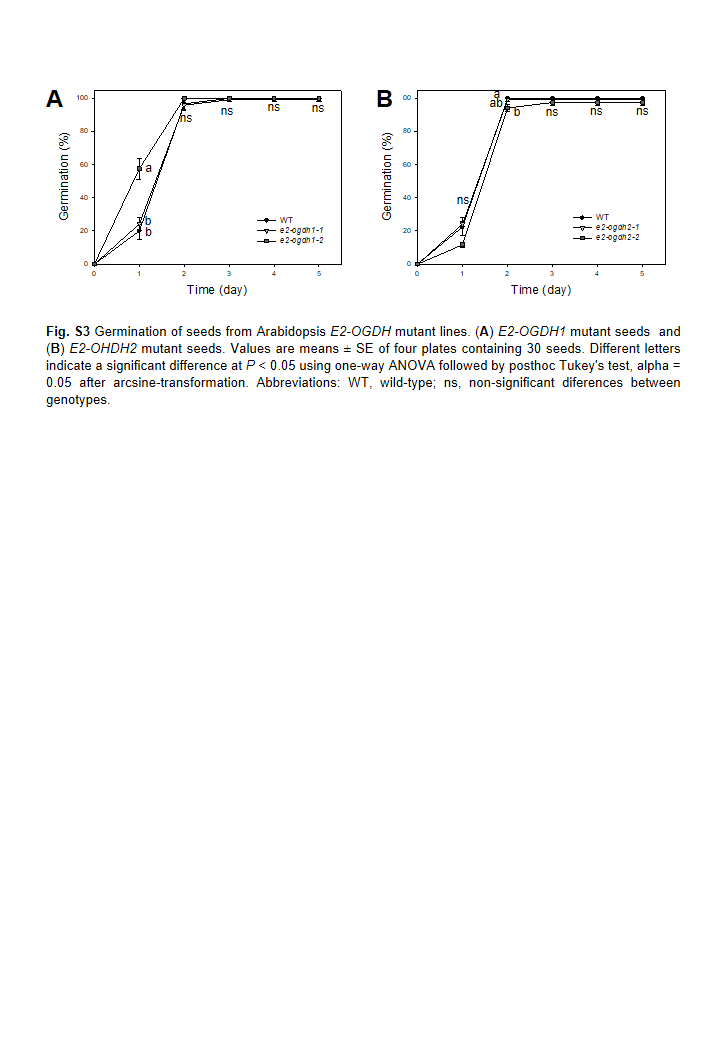

Supplement: pcab036_Supp [file pcab036_supp.zip › pcp-2020-e-00137-File012.TIF]

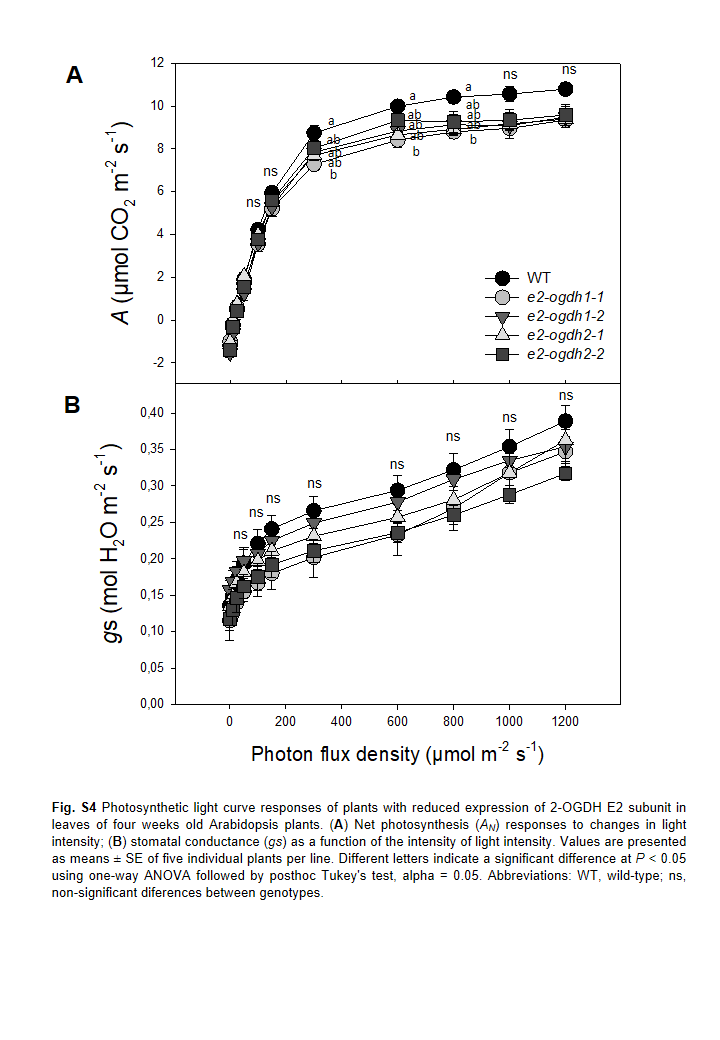

Supplement: pcab036_Supp [file pcab036_supp.zip › pcp-2020-e-00137-File013.tif]

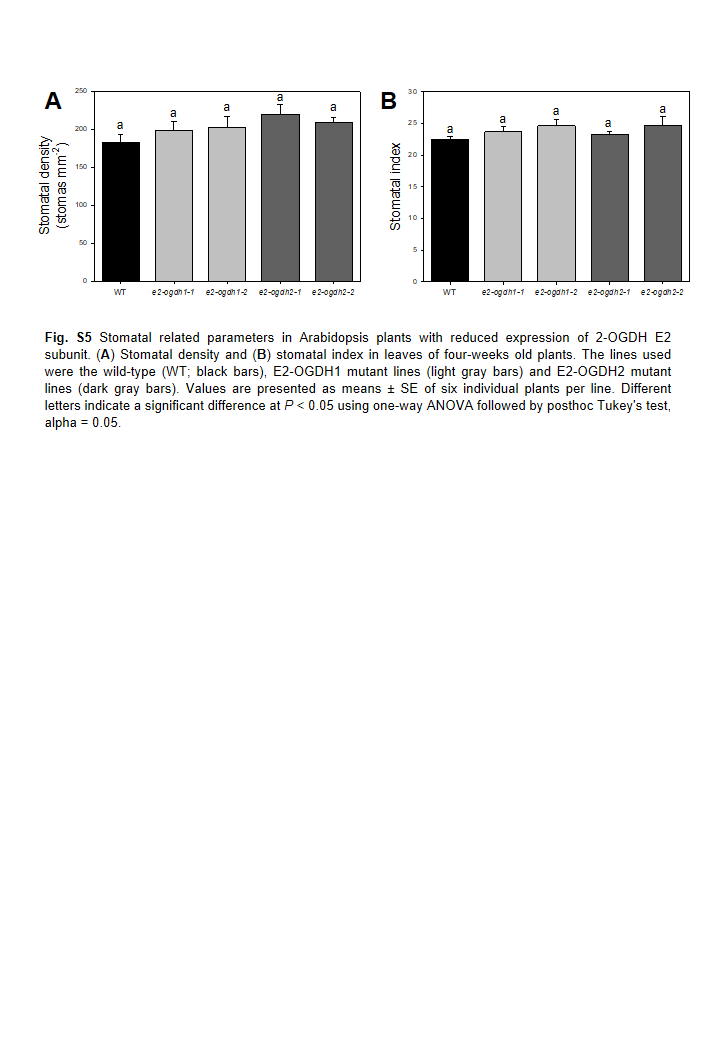

Supplement: pcab036_Supp [file pcab036_supp.zip › pcp-2020-e-00137-File014.TIF]
